# Transcriptomic Profiling Reveals Key Genes Underlying Cold Stress Responses in Camphora

**DOI:** 10.3390/life15020319

**Published:** 2025-02-19

**Authors:** Bowen Shi, Linlin Zheng, Yifeng Wang, Qirui Wang

**Affiliations:** College of Landscape Architecture and Art, Henan Agricultural University, Zhengzhou 450002, China; xek1206@henau.edu.cn (B.S.); w185380923@163.com (Y.W.)

**Keywords:** Camphora, cold resistance, transcriptomics, cold stress, photosynthesis, oxidative stress

## Abstract

The genus Camphora encompasses species of significant ecological and economic importance, such as *C. parthenoxylon* and *C. officinarum*, which exhibit distinct phenotypic traits and stress responses. This study seeks to elucidate the molecular basis of cold tolerance through comparative transcriptomic analysis complemented by physiological characterization. RNA sequencing revealed 6123 differentially expressed genes between the two species, with enriched pathways related to cold stress, oxidative stress, carotenoid biosynthesis, and photosynthesis. Key genes, such as annexin D5, chlorophyll a/b-binding protein, early light-induced protein 1, 9-cis-epoxycarotenoid dioxygenase, were identified as critical regulators of frost resistance, photosynthetic efficiency, and carotenoid biosynthesis. Functional enrichment analyses highlighted the involvement of signal transduction, membrane stabilization, and secondary metabolism in adaptive responses. Physiological assays supported these findings, showing higher chlorophyll and carotenoid content and enhanced antioxidative enzyme activities in *C. parthenoxylon*. These results provide valuable insights into the genetic and biochemical mechanisms underlying stress adaptation in Camphora species and offer promising targets for enhancing resilience in economically valuable plants.

## 1. Introduction

The genus Camphora, which includes species such as *Camphora parthenoxylon* and *Camphora officinarum*, holds significant ecological and economic value due to its diverse applications, including the production of essential oils, medicinal properties, and adaptability to varying environmental conditions [1,2,3]. However, these species exhibit distinct phenotypic differences, particularly in leaf characteristics and their response to environmental stressors such as cold temperatures. *C. parthenoxylon* possesses larger, greener leaves that remain intact during winter, showcasing remarkable frost resistance [4]. In contrast, *C. officinarum* exhibits smaller, yellowed leaves that are more sensitive to cold stress [4]. Understanding the molecular mechanisms underlying these differences is critical for improving the resilience, which could have broader implications for environmental practices and climate change adaptation strategies.

Previous studies on frost tolerance in plants have highlighted the involvement of key molecular players, such as genes regulating cell membrane integrity, the accumulation of cryoprotectants (e.g., sugars and proteins), and the modulation of stress-responsive signaling pathways [5,6,7]. These mechanisms have been shown to help prevent cellular damage due to ice formation during freezing conditions. Additionally, the synthesis of antifreeze proteins (AFPs) has been identified as a crucial element of frost resistance, as these proteins inhibit ice crystal formation and stabilize cell structures under low temperatures [8,9,10]. Transcription factors, such as CBF/DREB1, play a pivotal role in regulating cold-responsive genes [11], which activate the expression of protective proteins and metabolites, thereby mitigating the effects of freezing. Investigating these cold-responsive signaling pathways provides valuable insights into how plants adapt to low temperatures.

In addition to frost tolerance, leaf characteristics such as size, shape, and color are also crucial for plant survival and productivity. The size and chlorophyll content of leaves are closely linked to the plant’s ability to photosynthesize efficiently. Chlorophyll biosynthesis and degradation are tightly regulated by a set of enzymes and metabolic pathways [12], which control chlorophyll levels and influence leaf coloration. Leaf yellowing often occurs under stress, particularly in cold conditions, as chlorophyll breakdown is accelerated during unfavorable environmental conditions [13]. Recent studies have identified specific genes involved in the regulation of leaf size and color [14,15]. These include genes that control cell division and expansion, as well as genes involved in the hormonal regulation of leaf senescence and chlorophyll degradation during stress events such as cold or drought.

Transcriptomic studies have proven to be powerful tools for understanding the genetic basis of complex traits like stress tolerance [16]. In species such as *Arabidopsis thaliana*, transcriptomic studies have identified large sets of genes responsive to cold stress, shedding light on the molecular pathways involved in freezing tolerance. Similar studies on other tree species, including Populus [17], have revealed the upregulation of genes associated with protective mechanisms such as cell wall modification, detoxification of reactive oxygen species (ROS), and the synthesis of cryoprotectants during cold stress. These findings suggest that cold tolerance in trees may involve shared molecular pathways, which are likely present in Camphora species as well.

This study seeks to fill the gap in our understanding of the molecular mechanisms underlying the distinct leaf characteristics and frost tolerance observed in *C. parthenoxylon* and *C. officinarum*. By conducting a comparative analysis of the transcriptomes of these species, this research aims to explore the molecular and biochemical foundations of these traits. Specifically, the study focusses on identifying differentially expressed genes (DEGs) that contribute to differences in leaf size, color, and frost resistance. By integrating these molecular findings with physical-chemical property analyses, this study offers a comprehensive view of how these plants respond to environmental stress and provide valuable insights for improving the resilience of Camphora species.

## 2. Materials and Methods

### 2.1. Plant Materials and Sample Collection

Leaves were collected from two tree species—*Camphora parthenoxylon* and *Camphora officinarum*—growing under natural conditions at the Research center of Henan Academy of Forestry Sciences, China. Both trees were growing under identical environmental conditions. Samples were specifically collected at the onset of winter, coinciding with the visible yellowing of *C. officinarum* leaves, which marks the beginning of the stress response. This timing is critical as it allows us to capture the initial genetic responses to cold stress. Fresh leaf samples were collected from each species, with three biological replicates per species (designated as Cp1, Cp2, Cp3 for *C. parthenoxylon* and Cc1, Cc2, Cc3 for *C. officinarum*). The samples were collected from trees in their natural state without any treatment interventions. The leaves were cleaned with distilled water to remove surface contaminants and then immediately processed for extraction and analysis.

### 2.2. Superoxide Dismutase (SOD) Activity

The measurement of SOD activity was measured using the nitroblue tetrazolium reduction method by assessing its capacity to impede the reduction of WST-8 by superoxide radicals (NM-W-0101, Norminkoda Biotechnology Co., Ltd. Wuhan, China), resulting in a colored compound that can be detected at 450 nm. The assay protocol involves homogenizing approximately 0.1 g of plant tissue in 1 mL of a 50 mM phosphate buffer (pH 7.8) containing 1 mM EDTA and 2% (*w*/*v*) polyvinylpyrrolidone to stabilize the enzyme and prevent proteolytic degradation. The homogenized sample was then centrifuged at 8000× *g* for 10 min at 4 °C to separate the clear supernatant used for the SOD activity assay.

The assay reaction was set up by adding the supernatant to a reaction mixture containing WST-8 and enzyme-specific reagents as outlined in the kit’s instructions. The mixture was incubated for 30 min at 37 °C to allow the reaction to proceed, after which the absorbance was measured at 450 nm. SOD activity was calculated based on the degree of inhibition of formazan dye formation using standard calibration curves provided by the enzyme assay kit. This method ensures accurate quantification of SOD activity in the tissue samples under study.

### 2.3. Carotenoids Content

Carotenoid concentrations were assessed through solvent extraction and spectrophotometric evaluation. Approximately 0.1 g of tissue was homogenized in 1 mL of extraction solution (80% acetone solution) under low-light conditions to avert pigment degradation. The homogenate underwent centrifugation, and the supernatant was subjected to analysis. Absorbance for green tissues was assessed at 470 nm, 649 nm, and 665 nm to account for chlorophyll interference. Carotenoid concentrations were determined using established formulas. Absorbance at 440 nm was utilized directly to ascertain carotenoid concentrations in non-chlorophyll samples.

### 2.4. Chlorophyll Content

The chlorophyll content was quantified by extracting pigments with a buffer and measuring absorbance at 649 nm and 665 nm using a spectrophotometer. Approximately 0.1 g of tissue was homogenized in the extraction buffer (80% acetone solution), centrifuged, and the supernatant was utilized for analysis. Concentrations of chlorophyll a, chlorophyll b, and total chlorophyll were determined utilizing Lambert-Beer’s law and established formulas.

### 2.5. Catalase (CAT) Activity

The activity of catalase (CAT) was assessed by quantifying the decomposition of hydrogen peroxide (H_2_O_2_) by the enzyme. Approximately 0.1 g of tissue was homogenized in 1 mL of extraction buffer (50 mM phosphate buffer (pH 7.8) containing 1 mM EDTA and 2% (*w*/*v*) polyvinylpyrrolidone), centrifuged at 12,000× *g* for 10 min, and the supernatant was retrieved. The reaction mixture comprised the supernatant and H_2_O_2_, succeeded by the incorporation of ammonium molybdate to halt the reaction and generate a yellow complex. The absorbance of the complex was recorded at 405 nm. The CAT activity was determined using standard curves and specified equations.

### 2.6. Peroxidase (POD) Activity

The POD activity was evaluated using the guaiacol oxidation method based on the enzyme’s capacity to oxidize guaiacol in the presence of H_2_O_2_, resulting in the formation of a reddish product. Tissue samples (0.1 g) were homogenized in 1 mL of extraction buffer (50 mM phosphate buffer (pH 7.8) containing 1 mM EDTA and 2% (*w*/*v*) polyvinylpyrrolidone) and centrifuged at 12,000× *g* for 10 min. The reaction commenced with the addition of the supernatant, guaiacol, and H_2_O_2_ to the reaction mixture. The absorbance at 470 nm was recorded immediately upon reaction initiation and subsequently after 1 min. The variation in absorbance over time was utilized to determine POD activity.

### 2.7. Transcriptomic Analysis

The Tiangen DP441 polysaccharide and polyphenol RNA isolation kit (Tiangen Biotech, Beijing, China) was utilized to extract total RNA from the leaf tissues of both *C. parthenoxylon* and *C. officinarum*. An Agilent 2100 bioanalyzer was utilized to perform quality and integrity checks on the RNA.

RNA-seq libraries were constructed by Beijing Novogene Co., Ltd., Beijing, China, which utilized the All-In-One 5× RT MasterMix kit. These libraries were then sequenced on the Illumina NovaSeqTM 6000 platform. We performed paired-end sequencing on 36 libraries, with three biological replicates for each time point and condition. The sequencing was performed at a length of 150 base pairs.

The raw reads were filtered with Trimmomatic to ensure their quality, and the clean reads were aligned with the reference genome [1] with the help of HISAT2. Through the utilization of featureCounts, the levels of gene expression were measured in FPKM, which stands for fragments per kilobase per million. Differential expression analysis was carried out with DESeq2, with the cutoff for significant differential expression being set at |log_2_FC| ≥ 1 and FDR ≤ 0.05 when determining the significance of the difference.

### 2.8. Quantitative Real-Time PCR (qRT-PCR) Validation

Primer design and qRT-PCR conditions: nine DEGs were randomly selected for qRT-PCR validation. Primers were designed with the help of the Primer5 software (Table S1), and the BIO-RAD CFX 96 Touch Real-Time PCR system was utilized for the operation of the amplification process. As a point of reference within the study, the *CcamActin* (*Ccam12g01070*) gene was utilized.

Prerequisites for the qRT-PCR reaction: for each reaction, a reaction mixture of 10 μL was utilized. This mixture consisted of 5 μL of BlasTaq™ 2× qPCR MasterMix, 1 μL of cDNA, 0.3 μL of each primer (10 μM), and 3.4 μL of nuclease-free water. Three minutes of cycling at 95 °C was followed by forty cycles of 95 °C for fifteen seconds and then 60 °C for one minute. A method known as 2^−ΔΔCT^ was utilized to determine the relative expression levels.

### 2.9. Statistical Analysis

The data collected from physiological assays were subjected to Student’s *t*-test to verify the significance of difference between two genotypes. Results were expressed as mean ± standard deviation (SD). Differences were considered statistically significant at *p*-values less than 0.05. Similar was performed for qRT-PCR results.

## 3. Results

### 3.1. Morpho-Physiological Diferences Between C. parthenoxylon and C. ofcinarum

**Figure 1:**
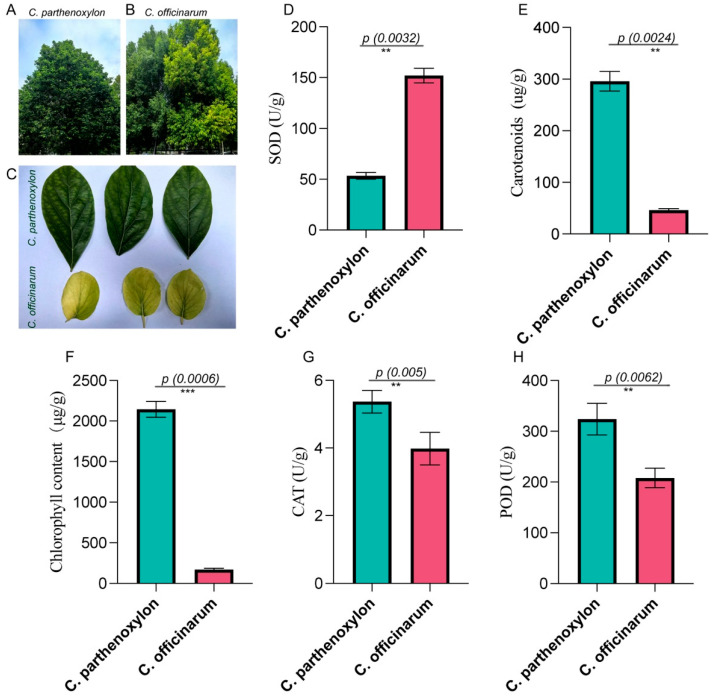
Morphological and physiological differences between *C. parthenoxylon* and *C. officinarum*. (**A**,**B**) Representative tree morphology of *C. parthenoxylon* (**A**) and *C. officinarum* (**B**). (**C**) Comparison of leaf morphology between *C. parthenoxylon* and *C. officinarum*, showing differences in leaf size and color. (**D**–**H**) Biochemical measurements comparing the two species: (**D**) SOD activity (U/g fresh weight); (**E**) carotenoid content (µg/g fresh weight); (**F**) total chlorophyll content (µg/g fresh weight); (**G**) CAT activity (U/g fresh weight); and (**H**) POD activity (U/g fresh weight). Bars represent mean values ± standard error (SE) from three biological replicates. *C. officinarum* shows significantly higher SOD activity, while *C. parthenoxylon* demonstrates higher carotenoid content, chlorophyll content, CAT activity, and POD activity. ** = significant at *p* < 0.01 and *** = significant at *p* < 0.001.

The morphological differences between *C. parthenoxylon* and *C. officinarum* were evident in their tree and leaf structures. *C. parthenoxylon* exhibited a dense canopy with dark green foliage (Figure 1A), while *C. officinarum* displayed a lighter green canopy and a more upright growth habit (Figure 1B). Leaf morphology also differed significantly between the two species, with *C. parthenoxylon* showing larger, darker green leaves, whereas *C. officinarum* had smaller, lighter yellow-green leaves (Figure 1C). These differences in leaf color and size suggest variations in chlorophyll and carotenoid content, which align with the physiological measurements observed in subsequent analyses.

The SOD activity, carotenoid content, total chlorophyll content, CAT activity, and POD activity were evaluated for *C. parthenoxylon* and *C. officinarum* leaf blades (Figure 1D–H). The mean SOD activity was significantly higher (*p*-value = 0.0032) in *C. officinarum* (152.08 U/g fresh weight) compared to *C. parthenoxylon* (53.39 U/g fresh weight). In contrast, the mean carotenoid content was markedly higher (*p*-value = 0.0024) in *C. parthenoxylon* (295.86 µg/g fresh weight) than in *C. officinarum* (46.29 µg/g fresh weight). Similarly, *C. parthenoxylon* exhibited a significantly higher (*p*-value = 0.0006) total chlorophyll content (2144.24 µg/g fresh weight) than *C. officinarum* (170.08 µg/g fresh weight). The CAT activity was slightly higher (*p*-value = 0.005) in *C. parthenoxylon* (5.37 U/g fresh weight) than in *C. officinarum* (3.98 U/g fresh weight), while the POD activity was also elevated in *C. parthenoxylon* (324.04 U/g fresh weight) compared to *C. officinarum* (207.89 U/g fresh weight). Despite the apparent differences in SOD activity between the two genotypes, the variability within *C. officinarum* contributed to a lack of statistical significance (*p* > 0.05). These results highlight the distinct biochemical profiles of the two species, with *C. parthenoxylon* generally demonstrating higher levels of photosynthetic pigments and enzymatic activities, except for SOD.

### 3.2. Transcriptome Profiling to Identify Key Biological Factors

RNA sequencing was performed to investigate the transcriptomic profiles of the samples using a next-generation sequencing platform with paired-end reads of 150 bp. Total RNA was extracted from biological replicates, and the integrity and quality of RNA were assessed using Nanodrop, agarose gel electrophoresis, and RIN value measurements. High-quality RNA samples with OD260/280 ≥ 1.8 and OD260/230 ≥ 1.0 were used for library preparation.

The sequencing quality metrics confirmed the high reliability and integrity of the data generated in this study (Table S2). Across all samples, the percentage of clean reads was consistently above 99.74%, with some samples achieving 100% clean reads post-filtering. The clean base yield ranged from approximately 222 million to 7.54 billion bases per sample, with an average GC content between 44.41% and 46.01%. Quality scores indicated high base-calling accuracy, with over 98.47% of bases achieving a Phred quality score of ≥Q30, and over 99.55% achieving ≥Q20. These metrics demonstrate the high-quality sequencing data and establish a robust foundation for downstream transcriptomic analyses.

Principal component analysis (PCA) was conducted to assess the variability among the samples (Figure S1). PC1 and PC2 explained 70.69% and 12.31% of the total variance, respectively. The samples formed two distinct clusters corresponding to the genotypes *C. parthenoxylon* and *C. officinarum*, indicating clear separation in transcriptomic profiles between the two species. This clustering highlights significant differences in their genetic expression patterns.

A correlation heatmap was generated to evaluate the relationships among biological replicates (Figure S2). The correlation coefficients ranged from 0.60 to 1.00, with intra-genotype replicates exhibiting higher correlation values (≥0.94) compared to inter-genotype replicates (≤0.73). This demonstrates high consistency within replicates of the same genotype and distinct transcriptomic profiles between the two species.

The heatmap generated using transcriptomic datasets (Figure S3) highlights the clustering patterns of gene expression across the samples. The samples are grouped into two distinct clusters, corresponding to *C. officinarum* and *C. parthenoxylon*, demonstrating clear transcriptomic differentiation between the two species. The normalized expression values were observed with distinct patterns observed for each genotype, indicating unique gene expression profiles and potential differences in biological functions and metabolic pathways. This clustering reflects the robustness of the data and consistency within biological replicates.

### 3.3. Differential Gene Regulation Contributing to Frost Resistance

**Figure 2:**
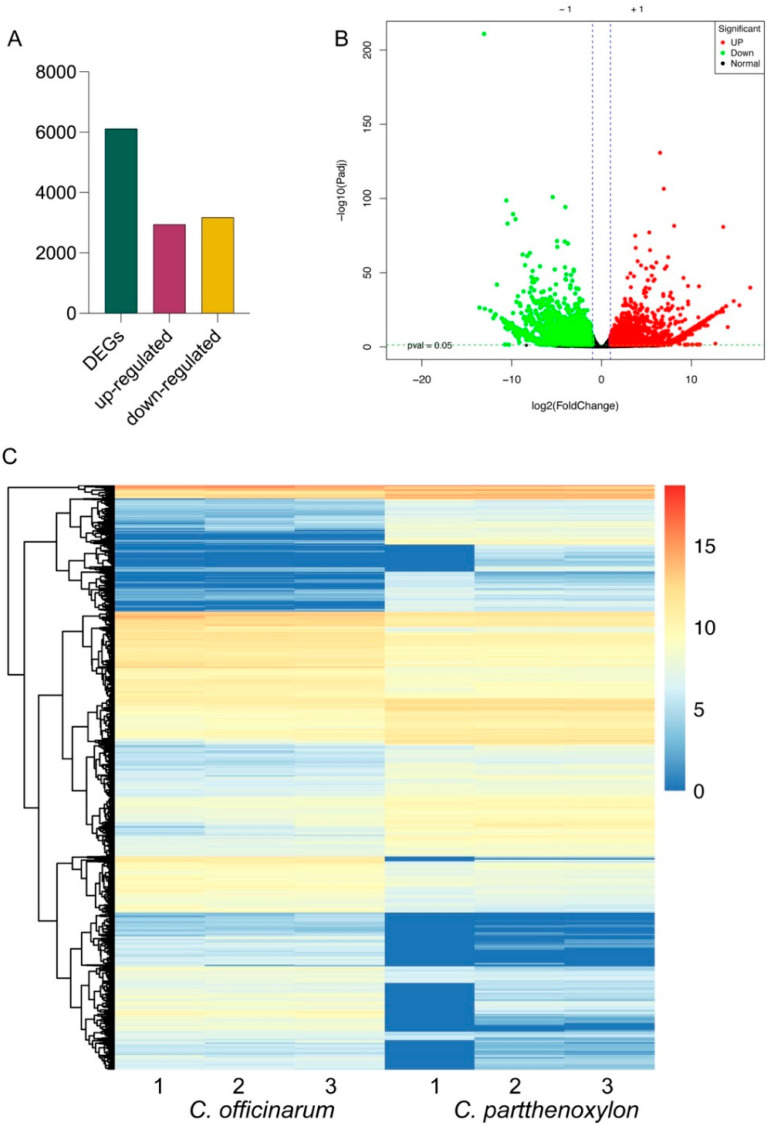
Differentially expressed genes (DEGs) between *C. parthenoxylon* and *C. officinarum*. (**A**) Total number of DEGs identified in the transcriptomic comparison, including the number of upregulated (2940) and downregulated (3183) genes. (**B**) Volcano plot depicting the distribution of DEGs based on log_2_ fold change (log_2_FC) and statistical significance (−log_10_ *p*-value). Red dots represent significantly upregulated genes, green dots represent significantly downregulated genes, and black dots indicate non-significant genes. (**C**) Heatmap showing hierarchical clustering of DEGs across biological replicates for both species. The clustering highlights distinct transcriptional profiles between *C. parthenoxylon* and *C. officinarum*. The color scale represents normalized gene expression levels, ranging from low (blue) to high (red).

In the transcriptomic comparison between *C. parthenoxylon* and *C. officinarum*, a total of 6123 DEGs were identified (Figure 2 and Table S3). Among these, 2940 genes were significantly upregulated, and 3183 genes were significantly downregulated (Figure 2A,B). These results indicate extensive transcriptional differences between the two species, with slightly more genes exhibiting downregulation compared to upregulation. This transcriptional variation underscores potential functional and metabolic differences between the two genotypes (Figure 2C).

**Figure 3:**
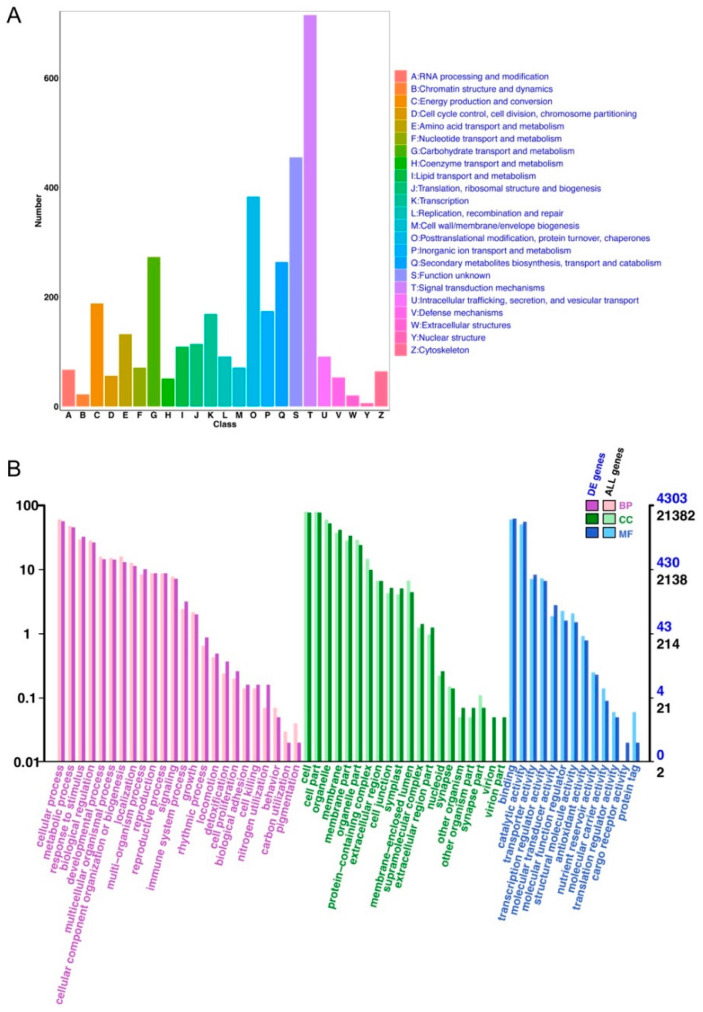
Annotation of differentially expressed genes (DEGs). (**A**) COG annotation of DEGs. The DEGs were classified into 25 functional categories according to the Clusters of Orthologous Groups (COG) database. The most enriched categories include “General function prediction only (R)”, “Signal transduction mechanisms (T)”, and “Posttranslational modification, protein turnover, chaperones (O)”. Each bar represents the number of DEGs assigned to a specific functional category. (**B**) GO annotation of DEGs. Gene Ontology (GO) classification is shown for biological process (BP), cellular component (CC), and molecular function (MF) categories. The most enriched terms in BP include “cellular process” and “metabolic process”. In CC, “cell part” and “membrane” were predominant, while in MF, “catalytic activity” and “binding” were highly represented. Bars indicate the number of DEGs associated with each GO term, with separate counts for DEGs and all annotated genes.

To further narrow down the genes associated with frost resistance, leaf senescence, and stress resistance, we performed annotation analysis for the DEGs. The Clusters of Orthologous Groups (COG) annotation classified the DEGs into 25 functional categories (Figure 3A), with notable enrichment in “Signal transduction mechanisms (T)”, “Posttranslational modification, protein turnover, chaperones (O)”, and “Defense mechanisms (V)”. These categories highlight the involvement of signaling pathways, protein stability, and defense responses, which are critical for frost resistance and stress adaptation. Gene Ontology (GO) annotation further supported these findings (Figure 3B), with biological process terms such as “response to stimulus”, “cellular process”, and “metabolic process” being highly enriched. In the molecular function category, “catalytic activity” and “binding” were predominant, indicating the roles of enzymes and molecular interactions in stress and senescence-related pathways. Cellular component terms such as “membrane” and “cell part” suggested that many DEGs are involved in membrane-associated functions, essential for sensing environmental changes and mediating protective responses. These functional annotations provide valuable insights into the molecular mechanisms underlying frost resistance, leaf senescence, and stress tolerance in the studied species.

The comparative transcriptomic analysis of *C. parthenoxylon* and *C. officinarum* revealed distinct expression patterns in genes associated with cold tolerance, photosynthesis, oxidative stress response, and secondary metabolism, providing insights into the molecular basis of their phenotypic differences.

DEGs identified in the dataset are related to processes such as frost resistance, stress resistance, leaf senescence, and leaf yellowing. These genes were selected based on their GO annotations and statistical significance. Many of these DEGs are involved in key stress response mechanisms, including oxidative stress resistance, calcium signaling, and cell wall fortification. For instance, genes like glutamate receptor-like protein (*Ccam05g01860*) and ferritin-3 (*Ccam09g00962*) play roles in calcium signaling and iron storage, which are crucial for coping with environmental stressors, including cold. Cinnamoyl-CoA reductase (*Ccam01g00516*), involved in lignin biosynthesis, contributes to strengthening the cell wall, helping the plant withstand physical stresses. Other genes, such as aconitate hydratase (*Ccam03g01028*) and NADP-linked oxidoreductase (*Ccam09g00597*), are part of metabolic pathways that mitigate oxidative damage, further enhancing the plant’s ability to endure frost and stress. Additionally, genes like senescence-related protein (*Ccam12g00115*) directly regulate leaf senescence, a process often triggered by stress, while early light-induced protein 1 (*Ccam10g00046*) is associated with light stress responses, potentially linked to leaf yellowing under certain environmental conditions. Together, these DEGs highlight the complex molecular mechanisms that plants employ to manage various stressors, including cold, oxidative damage, and senescence, providing valuable insights into their adaptive responses.

### 3.4. Differential Expression of Cold Tolerance Related Genes

**Figure 4:**
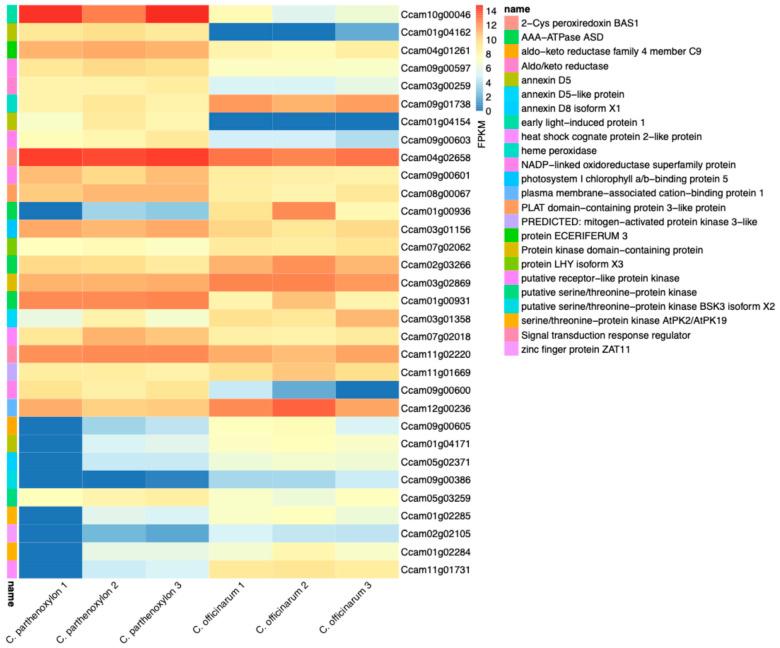
Differential expression of genes associated with cold tolerance in *C. parthenoxylon* and *C. officinarum*. The figure presents FPKM (fragments per kilobase of transcript per million mapped reads) values for genes implicated in cold tolerance mechanisms, including 2-Cys peroxiredoxin BAS1, annexin D5, heat shock cognate protein 2-like protein, mitogen-activated protein kinase 3-like, and zinc finger protein ZAT11, among others. Heatmap color intensity represents relative expression levels: warmer color (red) indicates higher expression, and cooler color (blue) represent lower expression.

The analysis of cold tolerance genes revealed significant differential expression, highlighting their roles in frost resistance and stress adaptation in *C. parthenoxylon* and *C. officinarum* (Figure 4 and Table S4). Among the most notable genes, *Ccam01g04154* and *Ccam01g04162*, both annotated as annexin D5, exhibited the highest upregulation with log_2_FC values of 11.35 and 9.65, respectively, indicating their essential roles in stabilizing cellular membranes and regulating calcium signaling under cold stress. Another important gene, *Ccam10g00046*, encoding early light-induced protein 1, chloroplastic-like protein, was strongly upregulated (log_2_FC: 7.39), emphasizing its function in photoprotection and adaptation to low-temperature environments. Additionally, *Ccam09g00600*, an NADP-linked oxidoreductase superfamily protein, showed significant upregulation (log_2_FC: 6.09), suggesting its involvement in managing oxidative stress by maintaining redox balance during cold exposure. In contrast, *Ccam01g00936*, encoding AAA-ATPase ASD, mitochondrial-like protein, exhibited marked downregulation (log_2_FC: −8.20), possibly reflecting a reduced role in mitochondrial activity to conserve energy for cold-specific stress responses. Similarly, *Ccam09g00386*, annotated as a putative serine/threonine-protein kinase BSK3, was downregulated (log_2_FC: −4.93), suggesting a shift in signaling priorities under cold conditions. Interestingly, *Ccam11g01731*, encoding a heat shock cognate protein 2-like protein, was also downregulated (log_2_FC: −4.48), potentially indicating a lower reliance on heat shock mechanisms in favor of cold-specific pathways. Lastly, *Ccam09g00603*, another NADP-linked oxidoreductase superfamily protein, exhibited upregulation (log_2_FC: 3.92), further highlighting the importance of redox balance in mitigating reactive oxygen species during cold stress. These findings underscore the critical roles of annexins, oxidoreductases, and light-induced proteins in enhancing cold stress tolerance, while also revealing a reprogramming of stress signaling and energy allocation mechanisms in response to low temperatures. Collectively, these results provide valuable insights into the molecular mechanisms underlying the cold tolerance observed in *C. parthenoxylon* and *C. officinarum*.

### 3.5. Differential Expression of Photosynthesis-Related Genes

**Figure 5:**
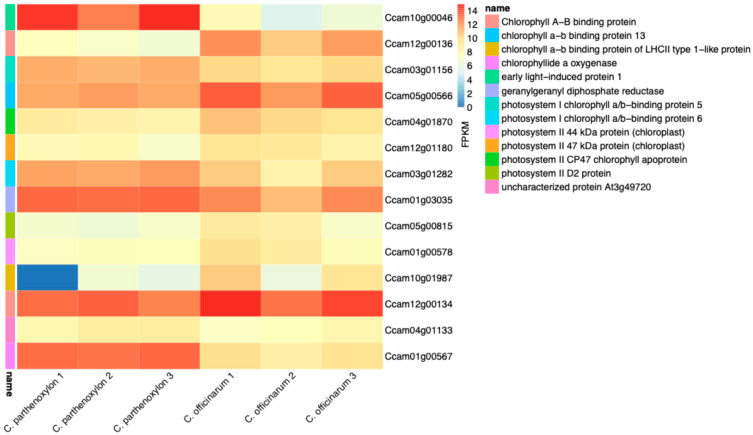
Differential expression of genes associated with photosynthesis in *C. parthenoxylon* and *C. officinarum*. The figure illustrates the FPKM values for genes involved in photosynthetic pathways, including chlorophyll a/b-binding proteins, photosystem I and II proteins, and chlorophyllide a oxygenase, among others. Samples are grouped by species and biological replicates, with three replicates for each species. Heatmap color intensity represents relative expression levels: warmer color (red) indicates higher expression, and cooler color (blue) represent lower expression.

The analysis of photosynthesis-related genes revealed significant differences in expression between *C. parthenoxylon* and *C. officinarum*, shedding light on their contrasting leaf characteristics and photosynthetic capacities (Figure 5 and Table S4). Among the most DEGs, *Ccam10g00046* (early light-induced protein 1) exhibited the highest upregulation (log_2_FC: 7.39), suggesting its critical role in photoprotection and adaptation to environmental stresses. Similarly, *Ccam01g00567* (chlorophyllide a oxygenase) was upregulated (log_2_FC: 3.78), emphasizing its importance in chlorophyll biosynthesis and maintenance of leaf greenness in *C. parthenoxylon*. In contrast, *Ccam12g00136* (chlorophyll a/b-binding protein) and *Ccam10g01987* (chlorophyll a/b-binding protein of LHCII type I) were significantly downregulated (log_2_FC: −4.71 and −3.77, respectively), particularly in *C. officinarum*, correlating with its diminished photosynthetic efficiency and smaller, yellowed leaves. Additionally, *Ccam03g01282* (photosystem I chlorophyll a/b-binding protein) showed moderate upregulation (log_2_FC: 1.74), indicating its role in maintaining photosystem I activity. Collectively, these findings highlight the differential regulation of photosynthesis-related genes as a key molecular mechanism underlying the distinct phenotypes of these two species, with *C. parthenoxylon* displaying enhanced photosynthetic performance and greater resilience under environmental stress.

### 3.6. Differential Expression of Carotenoid Biosynthesis Related Genes

**Figure 6:**
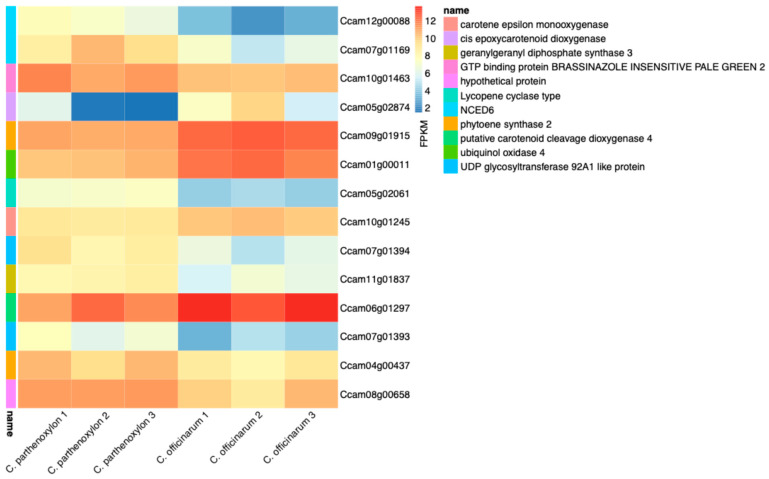
Expression levels of genes associated with carotenoid biosynthesis in *C. parthenoxylon* and *C. officinarum*. The figure presents the FPKM values for genes involved in key enzymatic steps of carotenoid biosynthesis, including carotene epsilon monooxygenase, cis-epoxycarotenoid dioxygenase, geranylgeranyl diphosphate synthase 3, phytoene synthase 2, and others. Samples are categorized by species and biological replicates, with clear distinctions between *C. parthenoxylon* and *C. officinarum* groups. Heatmap color intensity represents relative expression levels: warmer color (red) indicates higher expression, and cooler color (blue) represent lower expression.

The analysis of carotenoid biosynthesis-related genes revealed differential expression patterns that highlight their potential roles in regulating carotenoid metabolism and adaptation to environmental stress in *C. parthenoxylon* and *C. officinarum* (Figure 6 and Table S4). Among the most significantly expressed genes, *Ccam05g02874*, annotated as 9-cis-epoxycarotenoid dioxygenase, exhibited substantial downregulation (log_2_FC: −4.33), suggesting its reduced activity in carotenoid cleavage and possibly contributing to the modulation of abscisic acid (ABA) synthesis under specific stress conditions. Conversely, *Ccam12g00088*, another 9-cis-epoxycarotenoid dioxygenase gene, was strongly upregulated (log_2_FC: 4.18), highlighting its critical role in maintaining ABA synthesis, a key hormone in stress responses and stomatal regulation. Similarly, *Ccam07g01169*, also encoding 9-cis-epoxycarotenoid dioxygenase, showed notable upregulation (log_2_FC: 3.44), further emphasizing the importance of this pathway in mediating carotenoid turnover and stress adaptation. Additionally, *Ccam10g01463*, annotated as GTP-binding protein BRASSINAZOLE INSENSITIVE PALE-GREEN, exhibited moderate upregulation (log_2_FC: 1.04), pointing to its involvement in the broader network of stress-responsive signaling. These results underscore the dynamic regulation of carotenoid biosynthesis genes, particularly those encoding 9-cis-epoxycarotenoid dioxygenase, in contributing to the differential stress tolerance and physiological adaptations observed between the two species. The interplay of upregulated and downregulated genes within this pathway suggests a fine-tuned balance of carotenoid metabolism that supports stress resilience in varying environmental conditions.

### 3.7. Oxidative Stress Response and Secondary Metabolism Genes

Several genes associated with oxidative stress resistance were differentially expressed between the two species. In *C. parthenoxylon*, Aldehyde oxygenase (*Ccam04g01261*; log_2_FC: 3.07) was significantly upregulated, highlighting its role in mitigating reactive oxygen species (ROS) accumulation during cold stress. Additionally, early light-induced protein 1 (*Ccam10g00046*; log_2_FC: 7.39) was highly expressed, indicating its involvement in protecting photosynthetic machinery under stress. By contrast, *C. officinarum* displayed downregulation of oxidative stress-response genes, such as Caffeoylshikimate esterase (*Ccam11g01321*; log_2_FC: −7.34), reflecting its limited capacity to manage oxidative damage.

Differences in secondary metabolic pathways were also evident between the two species. In *C. parthenoxylon*, Phenylalanine ammonia-lyase (*PAL*) (*Ccam08g00432*; log_2_FC: 5.28) was significantly upregulated, suggesting a higher capacity for producing secondary metabolites such as flavonoids and lignin, which contribute to structural integrity and stress resistance. Conversely, *C. officinarum* showed downregulation of structural and defense-related genes, such as laccase (*Ccam09g00145*; log_2_FC: −3.65), which may explain its weaker cell wall integrity and heightened susceptibility to frost damage.

Overall, the upregulation of photosynthesis- and stress-response-related genes in *C. parthenoxylon* aligns with its phenotypic traits of larger, greener leaves and superior frost resistance. Conversely, the downregulation of these pathways in *C. officinarum* correlates with its smaller, yellowed leaves and increased sensitivity to cold stress. These results highlight the molecular foundations of their adaptive differences and offer valuable targets for further studies aimed at improving plant resilience to environmental challenges.

### 3.8. qRT-PCR Based Validation of Transcriptome Data

**Figure 7:**
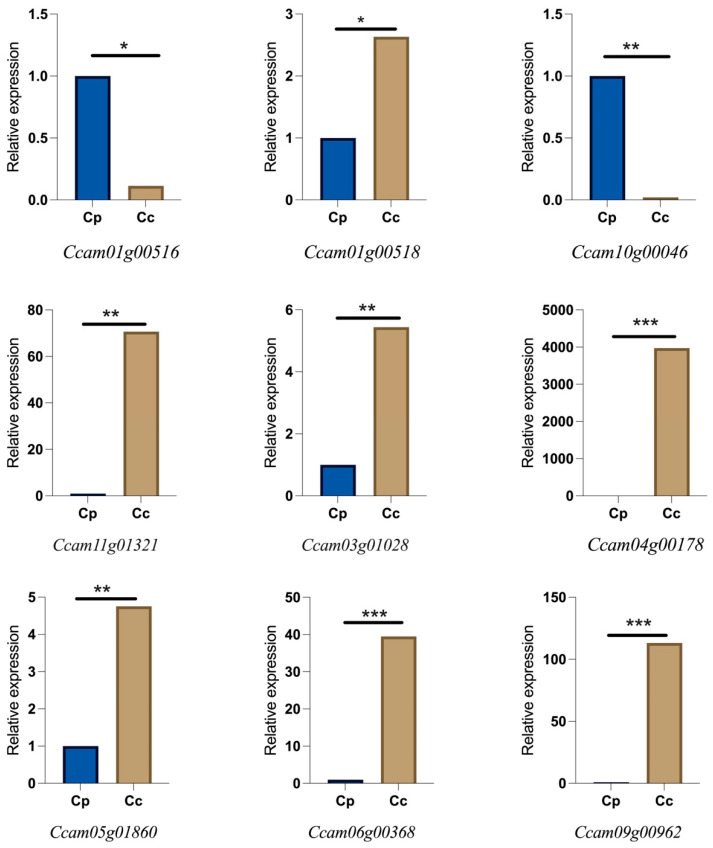
Quantitative RT-PCR-based validation of expression profiles for nine randomly selected genes from transcriptomic datasets of *C. parthenoxylon* (Cp) and *C. officinarum* (Cc). The bar plots represent the relative expression (2^−ΔΔCt^) levels of each gene in Cp and Cc, including *Ccam01g00516*, *Ccam01g00518*, *Ccam10g00046*, *Ccam11g01321*, *Ccam03g01028*, *Ccam04g00178*, *Ccam05g01860*, *Ccam06g00368*, and *Ccam09g00962*. The *y*-axis indicates relative expression, and the *x*-axis shows the two species. The significance of differences is highlighted based on the *p*-values calculated using Student’s *t*-test. * = significant at *p* < 0.05, ** = significant at *p* < 0.01 and *** = significant at *p* < 0.001.

To validate the expression profiles derived from transcriptomic datasets, nine genes were randomly selected, and their relative expression levels were quantified using qRT-PCR in *C. parthenoxylon* and *C. officinarum* (Figure 7). The qRT-PCR results confirmed the differential expression trends observed in the transcriptomic analysis, highlighting species-specific expression patterns. For instance, *Ccam10g00046* and *Ccam09g00962* exhibited significantly higher expression levels in *C. parthenoxylon* compared to *C. officinarum*, with fold changes exceeding 50. Similarly, *Ccam01g00518* and *Ccam05g01860* showed moderate but consistent upregulation in *C. parthenoxylon*. In contrast, genes such as *Ccam04g00178* and *Ccam06g00368* were more highly expressed in *C. officinarum* than in *Ccam01g00518*, further supporting the transcriptomic data. These findings support the reliability of the transcriptomic dataset and suggest that these genes may play roles in species-specific physiological or biochemical pathways.

## 4. Discussion

The findings of this study highlight significant molecular, biochemical, and phenotypic differences between *C. parthenoxylon* and *C. officinarum*. These differences highlight the complexity of their adaptations to environmental stresses, particularly cold temperatures. The differential expression of genes associated with frost resistance, photosynthesis, carotenoid biosynthesis, and oxidative stress responses, in relation to the physiological parameters examined, can elucidate the mechanisms underlying the distinct phenotypes of these organisms.

The frost tolerance exhibited by *C. parthenoxylon* can be attributed to the significant upregulation of genes such as annexin D5 (*Ccam01g04154* and *Ccam01g04162*), NADP-linked oxidoreductase superfamily proteins (*Ccam09g00600* and *Ccam09g00603*), and early light-induced protein 1 (*Ccam10g00046*). These genes play critical roles in maintaining cellular integrity and redox balance during cold stress. Annexins, for instance, stabilize cellular membranes under stress and mediate calcium signaling, a pathway widely recognized in plants for enhancing cold stress tolerance [18,19,20].

Previous studies have substantiated the importance of calcium signaling in cold stress adaptation. A recent study identified similar roles of annexins in mediating stress responses, supporting their significance in regulating cold-specific pathways [11]. Similarly, the enhanced expression of oxidoreductase genes aligns with findings in *Arabidopsis* [21,22,23] and Populus [17], where redox homeostasis was essential for coping with reactive oxygen species (ROS) during freezing.

The differential regulation of photosynthesis-related genes, such as chlorophyllide a oxygenase (*Ccam01g00567*) and chlorophyll a/b-binding proteins (*Ccam12g00136*, *Ccam10g01987*), sheds light on the physiological differences between the species. The enhanced photosynthetic efficiency in *C. parthenoxylon*, reflected by higher chlorophyll content and upregulated chlorophyll biosynthesis genes, aligns with its larger and greener leaves. These findings are consistent with research linking leaf pigmentation and photosynthetic capacity to stress resilience [12].

In *C. parthenoxylon*, the upregulation of genes such as Aldehyde oxygenase (*Ccam04g01261*) and Phenylalanine ammonia-lyase (*PAL*; *Ccam08g00432*) underscores its superior ability to mitigate oxidative damage and enhance secondary metabolite production. *PAL*, an essential enzyme in the phenylpropanoid pathway, is vital for the biosynthesis of lignin and flavonoids, which are recognized for their structural and protective functions under stress conditions [24,25,26]. The downregulation of these pathways in *C. officinarum* suggests a weaker defense mechanism against stress-induced cellular damage.

The contrasting regulation of 9-cis-epoxycarotenoid dioxygenase genes (*Ccam05g02874*, *Ccam12g00088*, *Ccam07g01169*) highlights the dynamic control of carotenoid metabolism in response to environmental stress. Carotenoids not only contribute to photoprotection but also serve as precursors for abscisic acid, a hormone central to stress responses [27,28,29]. The upregulation of these genes in *C. parthenoxylon* suggests a robust adaptive mechanism to environmental challenges, consistent with findings in other stress-resilient species.

The interaction between the pathways related to cold stress, oxidative stress, carotenoid biosynthesis, and photosynthesis is integral to a plant’s response to cold/frost. During cold stress, oxidative stress pathways are upregulated to manage increased reactive oxygen species production, preventing cellular damage [30]. Carotenoid biosynthesis contributes to this process by scavenging reactive oxygen species and facilitating the synthesis of abscisic acid (ABA), a stress hormone that enhances frost tolerance by modulating stomatal closure and other protective responses [31]. Concurrently, photosynthesis pathways adapt to the reduced temperatures and light conditions of frost scenarios. This includes the stabilization of photosynthetic complexes by chlorophyll a/b-binding proteins, ensuring optimal energy capture during stress. These pathways are interconnected through signaling molecules and transcription factors that regulate gene expression across different pathways, creating a coordinated defense mechanism that enhances the plant’s resilience to frost. This comprehensive network of responses illustrates the complex genetic adaptations of *C. parthenoxylon* and *C. officinarum* to their ecological niches, highlighting their distinct strategies for coping with environmental stress.

## 5. Conclusions

This study investigated the physiological and transcriptomic responses of *C. parthenoxylon* and *C. officinarum* to cold stress, elucidating how these responses contribute to their differential frost tolerance. Physiological analyses demonstrated that *C. parthenoxylon* displayed increased SOD and POD activities, whereas *C. officinarum* exhibited heightened CAT activity, signifying distinct strategies for mitigating cold stress. Variations in carotenoid and chlorophyll content further emphasized species-specific differences in photosynthetic efficiency. The transcriptomic analysis revealed that key genes such as annexin D5, chlorophyll a/b-binding protein, and 9-cis-epoxycarotenoid dioxygenase play pivotal roles in managing the physiological challenges posed by cold stress. These genes contribute to maintaining membrane stability, efficient photosynthesis under reduced light conditions, and the synthesis of abscisic acid, which collectively enhance cold endurance in *C. parthenoxylon*. Conversely, the relative downregulation of these genes in *C. officinarum* correlates with its increased vulnerability to cold stress. The results indicate that the two species utilize different mechanisms to manage cold stress, providing insights into their adaptive strategies and resilience.

## Data Availability

The datasets generated and/or analyzed during the current study are available at NCBI; Transcriptome sequencing data (under the project number: PRJNA1059809). Data will be publicly released upon acceptance of the manuscript.

## Supplementary Materials

The following supporting information can be downloaded at: https://www.mdpi.com/article/10.3390/life15020319/s1, Figure S1. Principal component analysis of transcriptomic data from *C. parthenoxylon* and *C. officinarum*. Figure S2. Correlation analysis of transcriptomic data from *C. parthenoxylon* and *C. officinarum*. Figure S3. Differential gene expression analysis between *C. parthenoxylon* and *C. officinarum*. Heatmap showing the 6123 DEGs identified through RNA sequencing. Table S1. Primer sequences used for qRT-PCR validation Table S2. Quality statistics for RNA sequencing. Table S3. Differential statistics of 6123 genes identified in comparison of *C. parthenoxylon* and *C. officinarum* Table S4. Expression profile of DEGs identified related to cold tolerance, photosynthesis, and carotenoid biosynthesis.
