# Supplementary figures and images for: Transcriptomic Profiling Reveals Key Genes Underlying Cold Stress Responses in Camphora
Source: Life (Basel). 2025 Feb 19;15(2):319. doi: 10.3390/life15020319 (PMC11857532; doi:10.3390/life15020319)

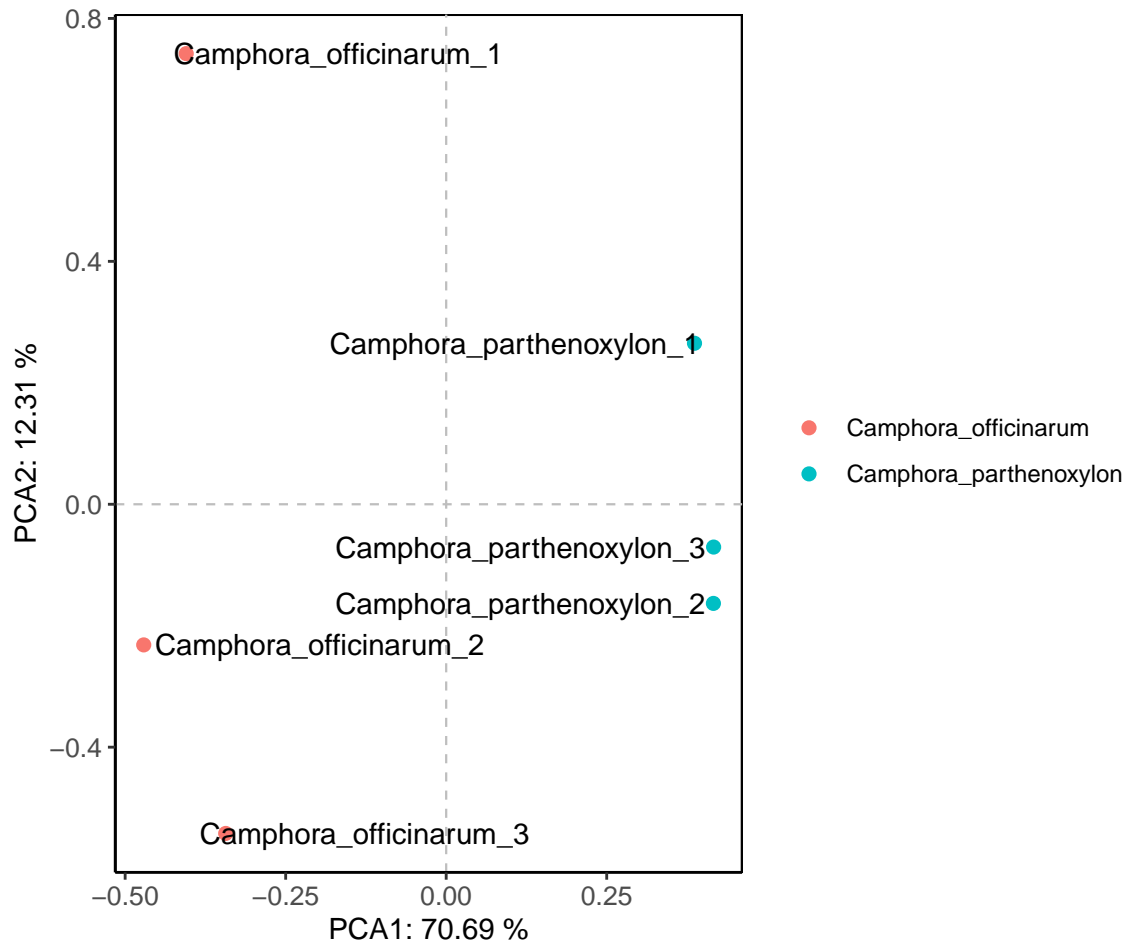

Supplement: Supplementary file 1 [file life-15-00319-s001.zip › Supplementary Material/Figure S1.pdf]

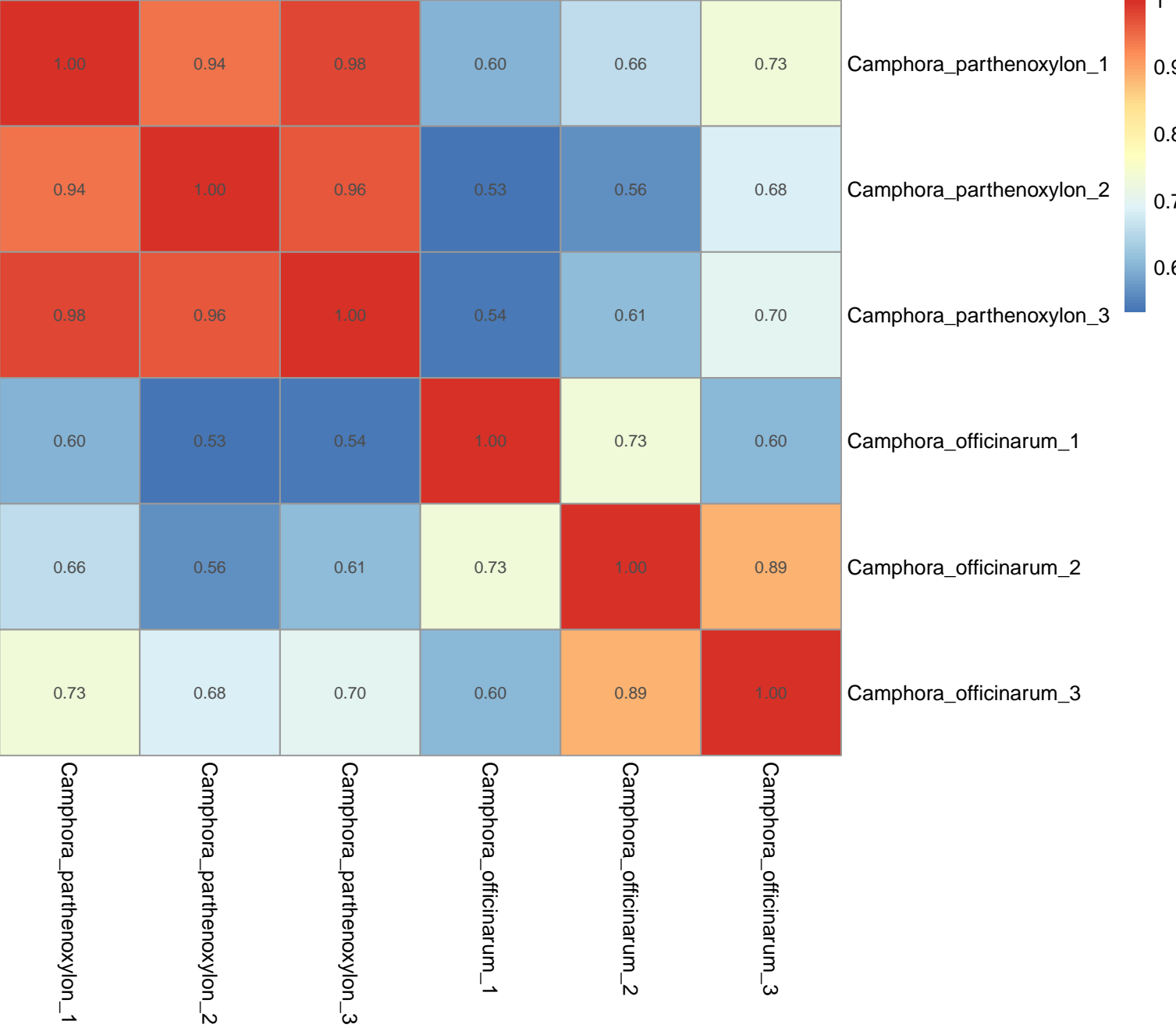

Supplement: Supplementary file 1 [file life-15-00319-s001.zip › Supplementary Material/Figure S2.pdf]

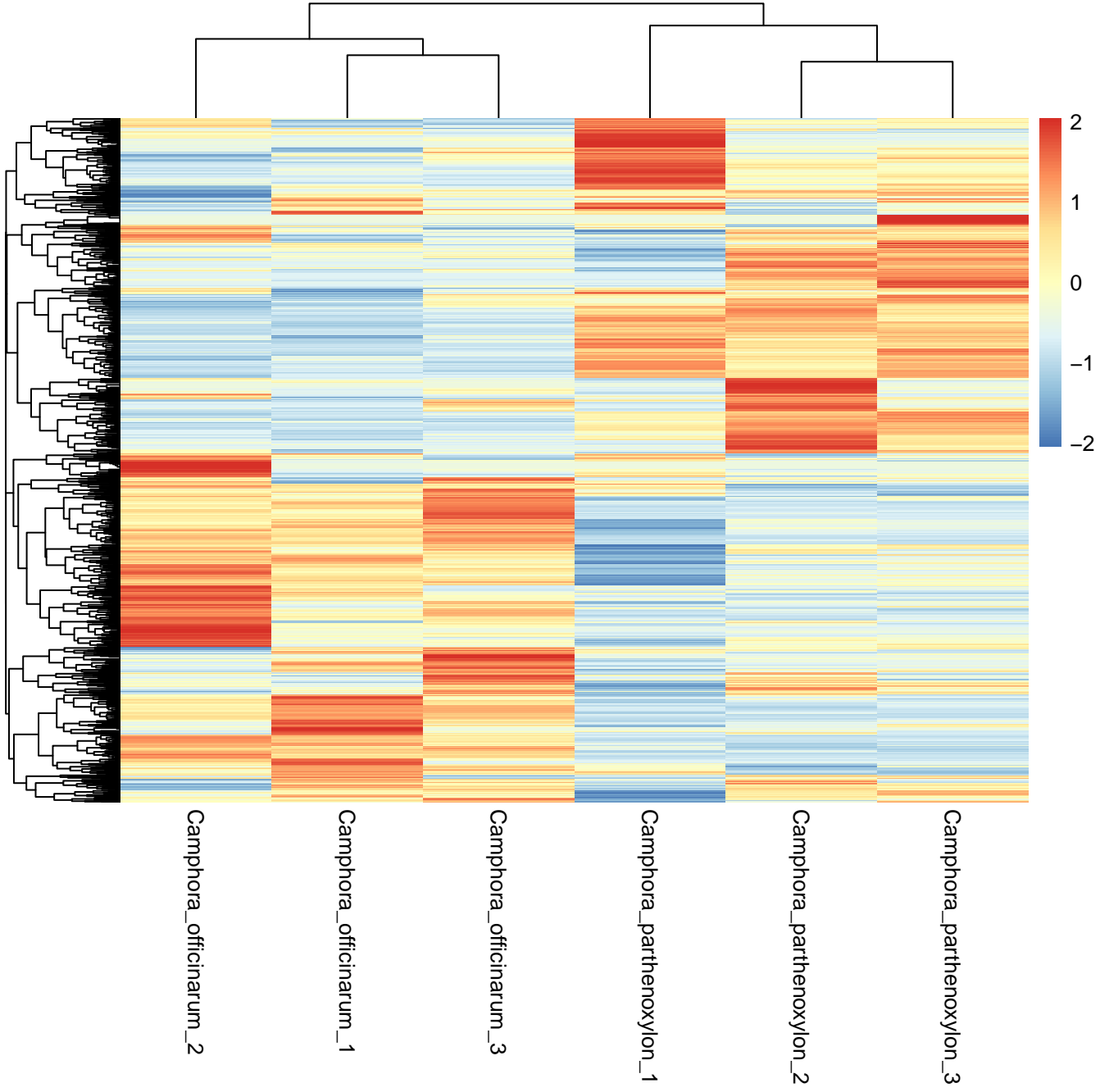

Supplement: Supplementary file 1 [file life-15-00319-s001.zip › Supplementary Material/Figure S3.pdf]
